# miR-204 Negatively Regulates HIV-Tat-Mediated Inflammation in Cervical Epithelial Cells via the NF-κB Axis: Insights from an In Vitro Study

**DOI:** 10.3390/cells15020117

**Published:** 2026-01-09

**Authors:** Kadambari Akolkar, Vandana Saxena

**Affiliations:** 1 Division of Immunology & Serology, ICMR-National Institute of Translational Virology & AIDS Research, Pune 411026, Maharashtra, India; akolkarkadambari093@gmail.com; 2 Division of Immunology & Serology, Savitribai Phule Pune University, Pune 411007, Maharashtra, India; 3 Faculty of Biological Sciences, Academy of Scientific and Innovative Research (AcSIR), Ghaziabad 201002, Uttar Pradesh, India

**Keywords:** cervical inflammation, HIV, miR-204-5p, NF-κB signaling

## Abstract

Despite antiretroviral therapy, HIV proteins, such as Tat, persist in tissues, driving chronic inflammation. Cervical inflammation in females not only accelerates HIV progression but also increases the risk of other STIs; hence, understanding the underlying factors/regulators is vital. However, Tat-induced cervical inflammation and its regulation are hitherto poorly understood, which we investigated using TZM-bl cells. Tat stimulation in these cervical epithelial cells significantly increased the expression of various inflammatory mediators, including cytokines (IL-1β, TNF-α, IL-6, IL-17a, GM-CSF), chemokines (MIP-1α, MIP-1β), adhesion molecules (ICAM-1, P-Selectin, E-Selectin), and ROS. Further upregulation of inflammatory mediators (NF-κB, IRAK-4) along with TLR7 was observed in Tat-stimulated cells. Interestingly, Tat stimulation decreased miR-204-5p expression in these cells, suggesting a role in regulating Tat-mediated inflammatory processes. Using a gain-of-function approach, we further observed that the overexpression of miR-204-5p reduced the expression of IL-1β, TNF-α, IL-6, MIP-1α, MIP-1β, ICAM-1, P-Selectin, and ROS in the Tat-stimulated TZM-bl cells, along with NF-κB, IRAK-1, and IRAK-4. Using Western blotting and luciferase assays, miR-204-5p was further shown to directly target NF-κB. Here, we report that HIV-1 Tat stimulation in cervical epithelial cells downregulates hsa-miR-204-5p, thereby activating the pro-inflammatory TLR7/NF-κB axis, highlighting its relevance to understanding mechanisms underlying cervical inflammation.

## 1. Introduction

Chronic immune activation, characterized by increased levels of pro-inflammatory cytokines and chemokines, is a pathognomonic feature of progressive HIV infection [1]. HIV acquisition and spread predominantly occur at mucosal surfaces, where mucosal inflammation further increases the risk of infection and transmission [2]. Among females, the cervicovaginal mucosa serves as the main portal of HIV and secondary transmission during heterosexual contact. Inflammation in the female genital tract creates an environment that favors HIV replication and establishment of a productive infection. Although combination antiretroviral therapy (cART) results in effective viral suppression, persistent immune activation and inflammation remain significant challenges. Even in the presence of cART, the early viral proteins, including HIV-1 Tat (Transactivator of Transcription), persist in tissues [3,4], contributing to chronic inflammation. Tat, an essential viral protein for HIV transcription and replication [5,6], is secreted from HIV-infected cells and perturbs both HIV-infected and bystander cells [7,8]. In context with the cervical microenvironment, it has been shown that extracellularly released HIV-1 Tat protein is internalized by the cervical cells and alters the intracellular milieu, resulting in increased progression of cervical carcinoma [9]. Further, several reports have highlighted that HIV proteins, including Tat, induce epithelial-to-mesenchymal transition (EMT) and promote the development of epithelial malignancies in HPV-16-immortalized anal, cervical, and oral epithelial cells [10]. Inflammation is one of the key drivers of EMT, where pro-inflammatory cytokines activate EMT, inducing transcription factors [11]. Cellular uptake of HIV-Tat has been shown to activate transcription factors, such as NF-κB [12], leading to pro-inflammatory cytokines, such as TNF-α, IL-6, CCL2, IL-8, and IL-1β, among others [6,13,14,15,16]. The role of HIV-1 Tat in inducing inflammation during neuroHIV and in the peripheral system has been widely studied, where Tat-induced NLRP3 inflammasome activation, releasing cytokines and chemokines in microglia [17,18] and enteric neuronal cells [19]. In contrast, less is known about HIV-Tat-mediated regulation of cervical inflammation and needs investigation. One of the important regulators of various cellular processes, including inflammation, is microRNAs. MicroRNAs (miRNAs/miRs) are a class of ~21–25 nucleotide-long, small non-coding RNA molecules that can downregulate gene expression by binding to a complementary sequence in the 3′ untranslated region (3′UTR) of target mRNAs. During HIV immunopathogenesis, miRNAs play multifaceted roles by regulating key aspects of immunopathogenesis, including viral replication, immune activation, and inflammation [20,21,22]. Numerous reports have indeed shown HIV-associated miRNA dysregulation that regulates inflammatory responses [23,24,25,26]. In microglia, HIV-1 Tat protein altered the profiles of various miRNAs and contributed to an increased inflammatory environment [27,28].

In our previous study, we observed dysregulation of miR-204-5p in cytobrush-derived cervical cells from HIV-infected females, accompanied by elevated levels of pro-inflammatory cytokines [29]. In a recent study, Kannan et al. also reported that miR-204-5p reduces HIV-Tat-mediated ferroptosis and the release of pro-inflammatory cytokines in microglial cells by targeting Acyl-CoA synthetase long-chain family member 4 (ASCL4) [30]. In other inflammatory conditions, miR-204-5p-mediated regulation of inflammation was facilitated by downregulating NF-κB [31,32]. Further, in various cancers, including gastric cancer [33], breast cancer [34], renal cell carcinoma [35], non-small cell lung cancer (NSCLC) [36], and glioma [37], miR-204 exerts regulatory roles. MiR-204-5p inhibited proliferation and invasion and induced apoptosis in cervical cancer cells by targeting different mediators, such as ATF2 and EphB2 [38,39,40]. Taken together, these findings underscore a probable role for miR-204 in regulating cellular functions in cervical cells, but whether it plays a role in the HIV-Tat-driven inflammatory response in these cells remains unknown. Given that during chronic HIV infection, Tat induces inflammation and could therefore be an important factor associated with the risk of cervical cancer, understanding the regulatory mechanism(s) associated with HIV-Tat-driven inflammation in cervical cells is warranted. In this study, we first examined the role of HIV-1 Tat in inducing inflammation in cervical epithelial cells under in vitro conditions, followed by an examination of the regulatory mechanisms involved. This study reveals that HIV-1 Tat-induced inflammation and oxidative stress are regulated by miRNA-204, which targets the NF-κB axis in HeLa cell-derived TZM-bl cells.

## 2. Materials and Methods

### 2.1. Reagents

Antibodies were acquired from the Cell Signaling Technology (Danvers, MA, USA): NF-ĸB (1:1000 dilution); IL-1β (1:1000 dilution); IRAK1 (1:1000 dilution); IRAK4 (1:1000 dilution); Anti-Rabbit IgG (1:2000 dilution). Cell culture growth medium, Dulbecco’s modified Eagle’s medium (DMEM), was purchased from Gibco (Waltham, MA, USA). Recombinant HIV-1 Tat protein and cellular ROS assay kit DCFDA/H2DCFDA were obtained from Abcam (Cambridge, UK). miRCURY LNA RT kit; miRCURY LNA miRNA PCR Assays for U6 and miR-204-5p; miRCURY LNA SYBR Green PCR Kit; HiPerfect Transfection Reagent were purchased from Qiagen (Hilden, Germany). TaqMan™ MicroRNA Reverse Transcription Kit and TaqMan™ MicroRNA assays for miR-204-5p and RNU-44 were purchased from Applied Biosystems (Waltham, MA, USA). miRIDIAN miRNA-204-5p mimic/inhibitor, miRIDIAN miRNA mimic negative control were procured from Dharmacon (Lafayette, CO, USA). TRIzol reagent and magnetic bead-based multiplex assay (ProcartaPlex) kit were from Invitrogen (Waltham, MA, USA). PowerUP SYBR Green Master Mix was procured from Applied Biosystems (Waltham, MA, USA).

### 2.2. RNA Extraction from Human Cervical Epithelial Cell Line

TZM-bl, a HeLa cell-derived human cervical epithelial cell line, was used in this study (NIH AIDS Research and Reference Reagent Program). TZM-bl cells were cultured in Dulbecco’s modified Eagle’s medium (DMEM; Gibco, Waltham, MA, USA) supplemented with 10% fetal bovine serum (FBS; Gibco, Waltham, MA, USA), 200 μg/mL L-glutamine, and 100 U/mL penicillin–streptomycin at 37 °C with 5% CO_2_.

For assay setup, 50,000 cells were stimulated with 50 ng recombinant HIV-1 Tat protein (Abcam, UK) for 24 h unless otherwise stated, followed by harvesting the cells and the supernatant. Supernatants were stored at −80 °C until further use in ELISA/bioplex assay. Cells were stored in TRIzol reagent (Invitrogen, Waltham, MA, USA), and RNA was isolated as per the manufacturer’s instructions. The quality of the samples was evaluated by estimating the A260 nm/A230 nm and A260 nm/A280 nm ratios for phenol and protein contamination, respectively, using a NanoDrop (DeNovix, Wilmington, DE, USA USA). Further, 250 ng of RNA was used for cDNA synthesis (Takara, California, USA), followed by real-time PCR.

### 2.3. Real-Time qPCR (RT-qPCR) Analysis

Gene expression profiling of various cytokines (TNF-α, IFN-β, IL-1β, IL-6), transcription factors (NF-ĸB), and intermediate signaling molecules (IRAK1 and IRAK4) was carried out by RT-PCR using PowerUp SYBR Green Master Mix (Applied Biosystems^TM^, Waltham, MA, USA). The β-actin gene was used as a housekeeping gene to determine the relative expression of the gene of interest. PCR reactions were performed on an AB7500 Fast (Applied Biosystems^TM^). Table 1 illustrates the primer sequences used in this study. For the expression levels of miRNAs, miRCURY LNA miRNA Custom PCR Panels (Qiagen) and TaqMan™ MicroRNA assays (Applied Biosystem) were used in RT-qPCR, and the data were normalized to the reference gene (U6 snRNA/RNU44). The fold change was calculated by the 2^−ΔΔCt^ method.

### 2.4. Transfection Assays

TZM-bl cells were seeded into 48-well plates (50,000 cells per well) or six-well plates (1 × 10^6^ cells per well) for gene expression analysis by RT-PCR and protein analysis by Western blotting, respectively. Cells were transiently transfected with 50 ng of miRNA-204-5p mimic/miR-204-5p inhibitor using HiPerfect Transfection Reagent (Qiagen, Germany) according to the manufacturer’s instructions. Transfection with scrambled miRNA served as a control for the assay. Briefly, miRNA-204-5p mimic/inhibitor and a scrambled miRNA negative control (mock) were incubated with HiPerfect reagent in DMEM (without FBS) for 10 min. This complex was slowly added to the single-cell suspension and incubated in DMEM supplemented with 10% FBS for 16 h. Following transfection, cells were exposed to HIV-1 Tat (50 ng/mL) for another 24 h. Total RNA and protein were extracted for further investigations.

### 2.5. Western Blotting

A total of 1 × 10^6^ TZM-bl cells were seeded in a six-well plate and harvested after 24 h of Tat stimulation. Briefly, the control, Tat-treated, and miR-204-mimic-transfected cells were lysed using 300 µL of Pierce^TM^ RIPA buffer (Thermo, Waltham, MA, USA). Lysates were incubated at 4 °C for 30 min and then centrifuged at 12,000× *g* for 10 min at 4 °C. Bradford assay (Bio-Rad, Herculus, CA, USA) was used to determine the protein concentrations as per the manufacturer’s instructions. A total of 100 µg of soluble proteins was resolved in 10% sodium dodecyl sulfate-polyacrylamide gel electrophoresis (SDS-PAGE), followed by blotting onto a 0.2 µm Immun-Blot polyvinylidene fluoride membrane (Bio-Rad, USA). Then the membranes were blocked with 5% non-fat dry milk in 1× Tris-Buffered Saline with Tween-20 (TBS-T) buffer for 1 h at room temperature, washed three times with TBS-T, and incubated overnight with primary antibodies at 4 °C. After three washes, the membranes were incubated with a secondary antibody for 1 h at room temperature. Next, the protein signals were developed using Clarity Western ECL substrate (Bio-Rad, USA) and visualized on ChemiDoc XRS+ System (Bio-Rad). Each band intensity was normalized to the internal control, GAPDH, and the data were presented as relative fold change by using Image Lab software (version 3.0.1) analysis.

### 2.6. Pro-Inflammatory Markers’ Analysis

Cytokine concentrations within the culture supernatants were quantified utilizing the magnetic bead-based ProcartaPlex™ Multiplex Immunoassay, Human Inflammation Panel I (Invitrogen, USA), in accordance with the manufacturer’s protocol. Briefly, 50 µL of cell supernatants were incubated with 50× simplex beads in a 96-well plate for 2 h at room temperature on a shaker. Subsequently, after washing the beads, a detection antibody was added for 30 min at room temperature. Following the washing, the beads were incubated with Streptavidin-PE for another 30 min. The samples were resuspended in reading buffer and analyzed using the LABScan 100^TM^ xPONENT system. The concentration of cytokines was derived from a standard curve generated by the standard provided in the kit. Calculations were performed using a four-parameter logistic (4PL). For the concentrations that were below the detection limit, the lowest obtained value was considered.

### 2.7. Target Identification and Validation by 3′UTR Luciferase Assay

miRNA-mRNA target prediction was carried out using miRWalk. RNA hybrid server (accessed on 20 September 2024; https://bibiserv.cebitec.uni-bielefeld.de/rnahybrid) was used to predict miRNA-mRNA interactions [48]. The NF-κB’s 3′UTR sequence was PCR-amplified with primers Fq-3′ CTAACTAGTCCTGCTGACAATTT-5′ and Rq-3′TATCAAGCTTTAAGCAACCTCATC-5′ and cloned downstream of the luciferase gene in the pMIR-REPORT luciferase vector. HEK 293 cells were transfected with reporter plasmid (500 ng) and miR-204-5p or scrambled oligonucleotide (gifted by Dr. Rajesh Gacche, Savitribai Phule Pune University, Department of Biotechnology, Pune, India) using jetPRIME (Polyplus, S.A, Illkirch, France) transfection reagent. After 24 h, cells were lysed using the Bright-GloTM Luciferase Assay System (Promega, Madison, WI, USA), and luciferase activity was measured on a PerkinElmer Victor X2 Multilabel Microplate Reader (Waltham, MA, USA).

### 2.8. Reactive Oxygen Species Production Analysis by DCFDA/H2DCFDA

To assess ROS production, TZM-bl cells were resuspended in DCFDA/H2DCFDA solution (Abcam, Cambridge, UK) at a concentration of 20 µM and incubated for 30 min at 37 °C in the dark. After incubation, the cells were stimulated with HIV-1 Tat protein for 15 min, 30 min, 60 min, and 120 min. Fluorescence was measured by flow cytometry. To evaluate the effect of hsa-miR-204-5p, ROS levels were measured in unstimulated control, Tat-treated, and hsa-miR-204-5p mimic-transfected or mock cells after 2 h of Tat stimulation.

### 2.9. NF-kB Nuclear Translocation Analysis by Fluorescence Microscopy

The translocation of NF-kB in TZM-bl cells was analyzed using a fluorescence microscope. Glass Coverslips (diameter 18 mm) were cleaned with ethanol, followed by UV exposure for 15 min, and then placed into a 12-well cell culture plate. In total, 0.1 × 10^6^ cells were seeded in a plate, grown overnight, and transiently transfected with either miRNA-204-5p mimic, miRNA-204-5p inhibitor, or scrambled miRNAs. They were then stimulated with 50 ng of HIV-1 Tat protein. Afterward, the cells were washed with PBS and fixed with fresh 4% paraformaldehyde solution for 20 min at room temperature. They were then washed three times with PBS and incubated for 15 min in blocking buffer containing 3% BSA and 0.1% Triton™ X-100 at room temperature. Subsequently, the cells were incubated with a primary monoclonal rabbit antibody against phospho-NF-kB (Cat. no. ab3033) for 1 h, diluted 1:1000 in antibody dilution buffer containing 3% BSA. The cells were washed three times with PBS and incubated for 30 min with secondary antibodies, goat anti-rabbit IgG CF@647 (2 µg), diluted in antibody dilution buffer (Biolegend, San Diego, CA, USA, Cat. no. 392111). After washing with PBS, they were incubated with DAPI (5 µg/mL; Invitrogen, Cat. No. D1306) for 5 min. Coverslips from a 12-well plate were carefully removed with the help of forceps and mounted on a glass slide with ProLong™ RapidSet™ Antifade Mountant (Thermo Fisher, Cat. no. P38931), and visualized using a fluorescence microscope (Zeiss Cell Discoverer 7.0, Carl Zeiss Microscopy GmbH, Jena, Germany, magnification 40).

### 2.10. Statistical Analysis

All the experiments were performed in triplicate and repeated three to four times. The data were expressed as mean ± SEM. An unpaired Student’s *t*-test was used to derive the statistical difference between two conditions/groups, while one-way ANOVA followed by post hoc Dunnett’s test was used to compare the difference between more than two experimental conditions. Statistical significance was determined using GraphPad Prism version 8.4.2 (San Diego, CA, USA). A *p*-value of ≤0.05 was considered to be statistically significant.

## 3. Results

### 3.1. HIV-1 Tat Primes the Inflammatory Responses in Cervical Epithelial Cells

**Figure 1:**
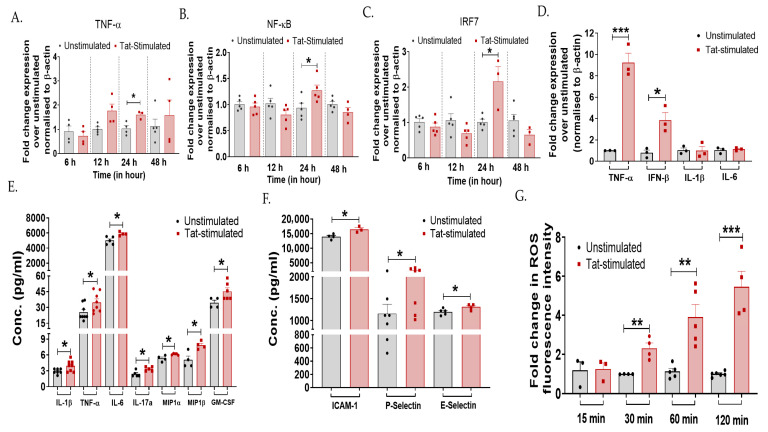
HIV-1 Tat increases the expression of inflammatory markers in the cervical epithelial cells. TZM-bl cells were stimulated with HIV-Tat (50 ng/mL) for different time intervals—6 h, 12 h, 24 h, and 48 h. This was followed by the RT-PCR analysis of (**A**) TNF-α (*p* = 0.02), (**B**) NF-ĸB (*p* = 0.04), and (**C**) IRF7 (*p* = 0.01) genes to determine the kinetics of inflammation. (**D**) TZM-bl cells exposed to HIV-1 Tat (50 ng/mL) for 24 h, mRNA levels (TNF-α, IFN-β, IL-1β, IL-6) and (**E**,**F**) protein levels of pro-inflammatory markers (IL-1β, TNF-α, IL-6, IL-17a, MIP-1α, MIP-1β, and GM-CSF, ICAM-1, P-Selectin, E-Selectin) were analyzed by RT-PCR and magnetic bead-based multiplex immunoassay, respectively. (**G**) TZM-bl cells were treated with H2DCFDA for 30 min, followed by Tat stimulation at varying time points (15 min, 30 min, 60 min, and 120 min). ROS activity was measured by flow cytometry. Fold change mRNA expression of Tat-stimulated samples was calculated over non-stimulated cells (control). All the experiments included a minimum of three replicates. Statistical analysis was performed by using the unpaired *t*-test. * indicates *p* < 0.05, ** indicates *p* < 0.01, *** indicates *p* < 0.001.

The HIV-Tat protein has been shown to induce pro-inflammatory factors in various cells, including monocytes, microglia, and enteric neurons [16,17,18,19]. However, its role in driving inflammatory processes in the cervical cells is largely undefined. Further, HIV-Tat has been shown to promote EMT in cervical cells, resulting in cancer progression. Since cervical inflammation facilitates EMT, here we examined the role of HIV-Tat in inducing inflammation in the cervical epithelial cells using in vitro assays. TZM-bl cervical epithelial cells were first stimulated with 50 ng/mL HIV-1 Tat for 6–48 h, followed by gene expression profiling of inflammatory mediators. A significant increase in mRNA expression of TNF-α (*p* = 0.02), NF-ĸB (*p* = 0.04), and IRF7 (*p* = 0.01) was noted at 24 h in the Tat-stimulated cells compared to the non-stimulated cells (Figure 1A–C). In subsequent assays, the 24 h time point post Tat stimulation was therefore used.

Furthermore, the inflammatory profiles of various mediators were determined using RT-PCR and a magnetic bead-based multiplex immunoassay. Tat-stimulation in TZM-bl cells increased the expression of TNF-α (*p* = 0.0007) and IFN-β (*p* = 0.015) gene, while no difference was noted in IL-1β and IL-6 gene expression between the Tat-stimulated cells and that of non-stimulated cells (Figure 1D). Secreted cytokine profile showed increased levels of IL-1β (*p* = 0.03), TNF-α (*p* = 0.04), IL-6 (*p* = 0.01), IL-17a (*p* = 0.01), MIP-1α (*p* = 0.01), MIP-1β (*p* = 0.01), and GM-CSF (*p* = 0.04) post Tat stimulation as compared to the unstimulated cells (Figure 1E). Other inflammatory markers, such as intracellular adhesion molecule (ICAM)-1 (*p* = 0.02), P-Selectin (*p* = 0.04), and E-Selectin (*p* = 0.04), were also significantly upregulated upon Tat stimulation (Figure 1F). Since one of the direct consequences of the inflammatory process is the production of reactive oxygen species (ROS), and Tat-mediated induction of ROS has previously been reported in microglial cells [48], contributing to neuroinflammation, we determined whether Tat induces ROS production in cervical cells using an in vitro DCFDA assay. A significant increase in ROS levels was observed gradually in Tat-stimulated cells from 30 min onwards (*p* < 0.05; Figure 1G). These in vitro findings collectively suggest that HIV-1 Tat primes the release of inflammatory mediators in the cervical epithelial cells.

### 3.2. Tat-Mediated Inflammatory Responses Involved TLR7/NF-ĸB Signaling in Cervical Cells

**Figure 2:**
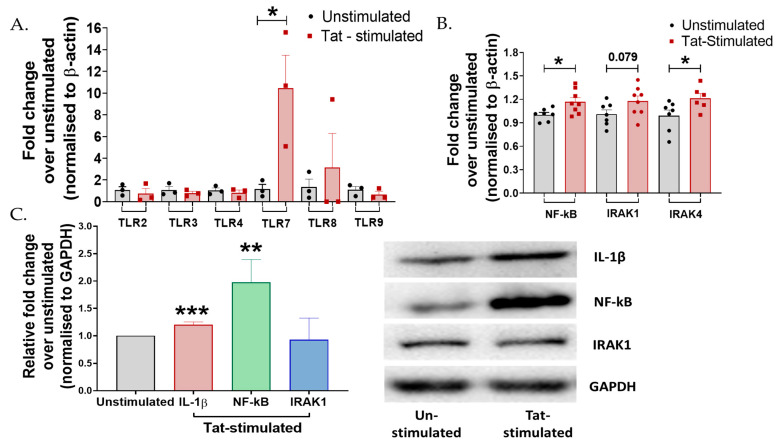
Tat mediates inflammatory responses via TLR7 cascading in cervical cells. (**A**) TZM-bl cells were stimulated with 50 ng Tat for 24 h. mRNA expression of TLR-2, 3, 4, 7, 8, and 9 was analyzed by RT-PCR. (**B**) mRNA levels of intermediates of the TLR7 (*p* = 0.01) pathway, IRAK1 (*p* = 0.07), IRAK4 (0.04), and NF-ĸB (0.02) were analyzed by RT-PCR. (**C**) Protein levels of intermediates of the TLR7 pathway, IRAK1 (*p* > 0.05), NF-ĸB (*p* = 0.004), and pro-inflammatory cytokine IL-1β (*p* < 0.001) were analyzed by Western blotting in Tat-stimulated TZM-bl cells. Fold change was calculated over the unstimulated control. All experiments included at least three replicates. Statistical analysis was performed by using the unpaired *t*-test. * indicates *p* < 0.05, ** indicates *p* < 0.01, *** indicates *p* < 0.001.

TLRs are known to stimulate expression of pro-inflammatory cytokines and induce inflammatory responses during the majority of infections [49,50]. Moreover, to explore the mechanism of Tat-mediated cervical inflammation, we examined the involvement of TLRs, intermediate proteins (IRAKs), and transcription factor (NF-ĸB) by RT-PCR and Western blotting. It was observed that among the various TLRs examined, TZM-bl cells exhibited the increased expression of the TLR7 gene to >8-fold post-Tat-stimulation (*p* = 0.01) than those of unstimulated cells (Figure 2A). Furthermore, increased expressions of NF-ĸB (*p* = 0.02), interleukin-1 receptor-associated kinase IRAK1 (*p* = 0.07), and IRAK4 (*p* = 0.04) were also observed in Tat-stimulated cells (Figure 2B) than in the unstimulated cells. In addition to unstimulated cells, heat-inactivated HIV-Tat was also used as a negative control. Indeed, TZM-bl cells did not elicit a measurable response to heat-inactivated Tat (Figure S1).

Protein expression analysis using Western blotting showed that HIV-1 Tat significantly increased the expression of IL-1β (*p* < 0.001) and NF-ĸB (*p* = 0.004), no significant change was observed in the expression of IRAK1 (*p* > 0.05) in the TZM-bl cells compared to the unstimulated controls (Figure 2C), suggesting the plausible role of TLR7/NF-ĸB signaling during Tat-mediated inflammatory responses in these cells.

### 3.3. miR-204-5p Regulates Tat-Mediated Inflammation by Targeting NF-κB in the Cervical Cells

**Figure 3:**
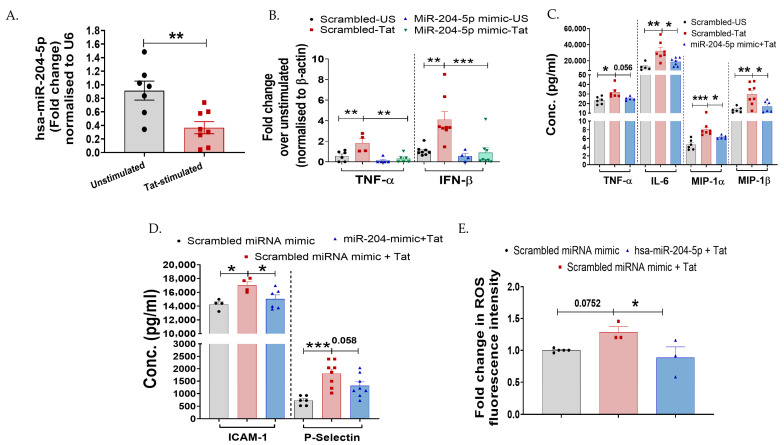
miR-204-5p regulates HIV-1 Tat-mediated inflammatory responses in cervical epithelial cells. (**A**) Expression of hsa-miR-204-5p in Tat-stimulated TZM-bl cells was examined over unstimulated control by RT-PCR (*p* = 0.005). (**B**) Effect of overexpression of hsa-miR-204-5p (50 nm, 24 h) on cervical cells during Tat-induced inflammatory cytokines was examined at mRNA levels (TNF-α and IFN-β by RT-PCR and (**C**,**D**) at protein levels (TNF-α, IL-6, MIP-1α, and MIP-1β) by using magnetic bead-based multiplex assay. Scrambled miRNA control (mock) was used as a control. (**E**) The effect of overexpression of hsa-miR-204-5p on ROS induction was examined in Tat-stimulated TZM-bl cells over a scrambled miRNA control (mock). All the experiments included a minimum of three replicates. Statistical analysis was performed by using the unpaired *t*-test and one-way ANOVA, followed by post hoc Dunnett’s test. * indicates *p* < 0.05, ** indicates *p* < 0.01, *** indicates *p* < 0.001.

Since we noted that HIV-Tat induces inflammation in cervical cells, we next sought to examine the regulatory factors involved. Previous reports showed that miR-204-5p is a negative regulator of inflammation [32,49]. In the microglial cells [30], hsa-miR-204-5p has previously been shown to regulate Tat-induced inflammatory processes. Further, its downregulation has been associated with metastasis of various cancers, including cervical cancer [38,50,51]. Hence, we first determined whether Tat stimulation alters miR-204-5p expression in vitro in cervical epithelial cells. A significant downregulated expression of miR-204-5p was noted in the TZM-bl cells post Tat stimulation (*p* = 0.005) than in the non-stimulated cells (Figure 3A).

**Figure 4:**
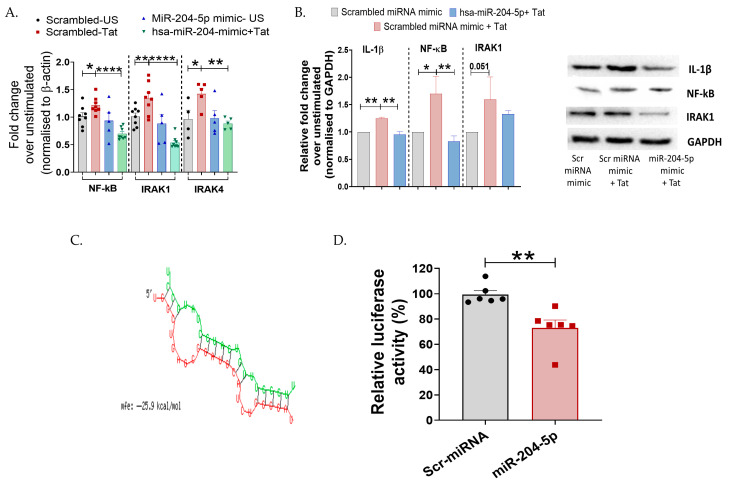
miR-204-5p targets NF-ĸB during HIV-1 Tat-mediated inflammatory responses in TZM-bl cervical epithelial cells. (**A**,**B**) mRNA expression and protein levels of transcription factor NF-ĸB, intermediate molecules IRAK1 and IRAK4, and pro-inflammatory cytokine IL-1β were analyzed by RT-PCR and Western blotting in Tat-stimulated TZM-bl cells. (**C**) Bioinformatics analysis identified complementarity between the seed sequence of miR-204-5p and its target in the 3′UTR region of NF-ĸB, with a free binding energy of −25.9 kcal/mol calculated using the RNA hybrid server. (Red: sequence of hsa-miR-2045p; Green: Target mRNA). (**D**) Luciferase reporter assay showing direct targeting of miR-204-5p with NF-ĸB. 3′UTR from NF-ĸB was cloned downstream of a luciferase reporter, then luciferase activity was measured following co-transfection with miR-204-5p or a scrambled (Scr) miRNA control. Decreased luciferase activity indicates direct targeting of the miR with that 3′UTR. The luciferase data were normalized to β-galactosidase RLU. All the experiments included a minimum of three replicates. Statistical analysis was performed by using the unpaired *t*-test and one-way ANOVA, followed by post hoc Dunnett’s test. * indicates *p* < 0.05, ** indicates *p* < 0.01, **** indicates *p* < 0.0001.

To further investigate the potential role of miR-204-5p in cervical cells, TZM-bl cells were transfected with miR-204-5p mimic, while a scrambled miR-mimic was used as a mock. Transfection conditions were confirmed in independent experiments using concentrations of 50 nm miR-204-5p and scrambled miR-mimic (mock) for 24 h. Cells transfected with 50 nM miR-204-5p mimic significantly increased the expression of miR-204-5p at 24 h (~53-fold; *p* < 0.0001) than mock-transfected cells (Figure S2). Role of miR-204-5p during HIV-Tat-induced cervical inflammation was assessed by overexpressing miR-204-5p in the TZM-bl cells, followed by quantifying the expression profile of various cytokines post Tat-stimulation using RT-PCR, magnetic bead-based multiplex assay, or Western blotting. It was found that TZM-bl cells transfected with miR-204-5p mimic had a significantly reduced expression of TNF-α (*p* = 0.002) and IFN-β (*p* = 0.0009) genes post Tat stimulation as compared to the cells transfected with scrambled miR-mimic/mock (Figure 3B). It is noteworthy that TZM-bl cells transfected with miR-204-5p mimic alone did not increase the inflammatory mediators (TNF-α, IFN-β, IL-1β, NF-ĸB, IRAKs) as compared to the mock-transfected cells (*p* > 0.05; Figure 3 and Figure 4). In the multiplex cytokine assay, inflammatory markers—TNF-α (*p* = 0.056), IL-6 (*p* = 0.01), MIP-1α (*p* = 0.03), MIP-1α (*p* = 0.002) ICAM-1 (*p* = 0.04), and P-Selectin (*p* = 0.01)—were also downregulated in the miR-204-5p mimic-transfected cells upon Tat stimulation (Figure 3C,D) than the mock cells. Moreover, overexpression of miR-204-5p reduced Tat-induced ROS production (*p* = 0.03) compared with mock cells (Figure 3E). These findings collectively envisage that miR-204-5p negatively regulates HIV-1 Tat-induced inflammatory responses in the TZM-bl cells.

Given that miRNAs exert their activity by targeting specific proteins in the signaling cascades, we further aimed to understand the mechanism of miR-204-5p-mediated regulation of inflammation in the cervical cells post HIV-Tat stimulation. Both IRAK-4 and NF-ĸB are intermediate molecules involved in TLR-mediated inflammatory cascades; hence, we examined their expression profiles. Since in our previous assay (Figure 2B), we observed upregulation of these mediators post-Tat-stimulation, we next sought to determine whether miR-204-5p exerts any regulatory role on these intermediate proteins using loss- and gain-of-function approaches. Interestingly, it was observed that miR-204-5p overexpression significantly decreased the expression of IRAK1 (*p* < 0.0001), IRAK4 (*p* = 0.05), and NF-ĸB (*p* < 0.0001) genes in the TZM-bl cells post Tat stimulation (Figure 4A). Using Western blotting, it was further noted that overexpression of miR-204-5p in these cells decreased IL-1β (*p* = 0.001) and NF-ĸB (*p* = 0.009) expression in response to Tat stimulation than that of mock cells (Figure 4B), while IRAK1 did not show any significant difference (*p* > 0.05). We also checked the effect of miR-204-5p inhibitor on the expression of cytokines and intermediate molecules in Tat-stimulated TZM-bl cells. An increase in protein expression levels of NF-kB and IRAK-1 was observed in miR-204-5p inhibitor-transfected and Tat-stimulated TZM-bl cells. Although the expression levels were not significant (Figure S3).

To reveal the role of TLR7 in Tat-mediated inflammatory signaling in TZM-bl cervical cells, we treated the cells with the TLR7 inhibitor M5049 (25 nM). Interestingly, a significant reduction in the expression of IFN-β (*p* = 0.0007), NF-ĸB (*p* = 0.001), IRAK1 (*p* = 0.003), and IRAK4 (*p* = 0.0001) was observed (Figure S4). No significant change in TNF-α expression was observed.

Using in silico tools—bioinformatics analysis with miRWalk and RNA hybrid server—we identified NF-ĸB as a putative target of miR-204-5p and calculated the binding energy (−25.9 kcal/mol) of miRNA–mRNA, respectively (Figure 4C). To confirm NF-ĸB as a direct target of miR-204-5p, we used a 3′UTR luciferase reporter system. Specifically, the 3′UTR of NF-ĸB was cloned into the reporter plasmid downstream of the luciferase gene in the pMIR-REPORT vector, then co-transfected with miR-204-5p mimic. It was found that NF-ĸB is indeed a direct target of miR-204-5p, as evidenced by a significant decrease in luciferase activity (*p* = 0.003, 24 h) following transfection with the miR-204-5p mimic (Figure 4D). Overall, these findings suggest a potent role of miR-204-5p in regulating cervical inflammation mediated through the TLR/NF-ĸB axis in the TZM-bl cells.

**Figure 5:**
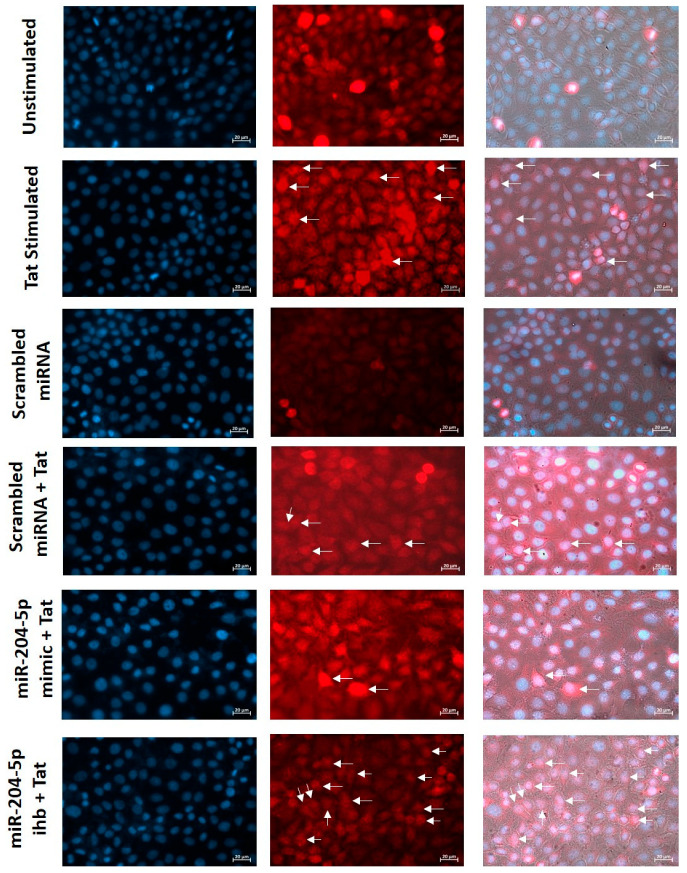
Expression of miR-204-5p regulates functional activity of NF-κB in Tat-stimulated cervical cells. Nuclear translocation of phospho-NF-κB was assessed by fluorescence microscope in Tat-stimulated TZM-bl cells and miR-204-5p mimic/inhibitor (ihb)-transfected cells before Tat stimulation. Images were captured using a fluorescence microscope (Zeiss Cell Discoverer 7.0, magnification 40). Nuclei were stained with DAPI (blue). Phospho-NF-kB was stained with CF@647 (red).

To provide functional evidence of NF-κB activation in Tat-stimulated TZM-bl cells, we assessed the nuclear translocation of phospho-NF-κB using a fluorescence microscope. The results indicated that Tat stimulation increased nuclear localization of phospho-NF-κB compared with non-stimulated cells. The reduction in phospho-NF-κB translocation was observed when cells were transfected with miR-204-5p mimic prior to Tat stimulation. Further, upon transfection with miR-204-5p inhibitor, an increased nuclear translocation of phospho-NF-κB was observed in Tat-stimulated TZM-bl cells compared to the cells transfected with scrambled miRNA (mock). These results provide direct evidence of NF-κB activity being altered in a miR-204–dependent manner in TZM-bl cells upon Tat stimulation (Figure 5).

## 4. Discussion

Among females, persistent cervical inflammation is a critical factor for increased viral shedding, thereby increasing the risk of HIV transmission. However, unlike peripheral and neuro-inflammatory responses, little is known about HIV mediated regulation of cervical inflammation. HIV proteins, particularly Tat and gp120, have been shown to induce chronic inflammation in microglia, monocytes, and epithelial cells, among others [17,52,53,54]. Although the cervicovaginal mucosa is an immunologically distinct site, HIV-Tat likely regulates inflammatory responses in cervical cells, as examined here using TZM-bl cells derived from HeLa cells under in vitro conditions.

In line with previous findings, our data showed that HIV-Tat drives inflammatory responses in cervical cells, resulting in the release of potent inflammatory cytokines (IL-1β, TNF-α, IL-6, IL-17a, and GM-CSF) [2,16,55]. We also observed that HIV-Tat upregulated the expression of chemotactic cytokines, including MIP-1α and MIP-1β, in TZM-bl cells. Elevated levels of these chemokines (MIP-1α and MIP-1β) have been detected in the genital secretions of HIV-infected women and have been linked to an increased risk of HIV acquisition [56]. GM-CSF has previously been shown to exert pro-inflammatory roles in many conditions [57,58,59]. During HIV infection, alveolar macrophages have been shown to increase the levels of GM-CSF, leading to pathogenic AIDS-associated interstitial lung disease [57]. Additionally, HIV proteins, including Tat, gp120, Nef, and Vpr, altered mitochondrial function and generated reactive oxygen species in brain tissues, leading to neuroinflammation [60,61,62]. In line with this, we also observed increased ROS levels in cervical epithelial cells post HIV-Tat stimulation, which might exacerbate inflammatory responses in these cells, together with other pro-inflammatory cytokines and chemokines. We also observed that Tat-stimulated cells increased the expression of adhesion molecules, including ICAM-1, P-Selectin, and E-Selectin. Tat-induced upregulation of these adhesion molecules has previously been reported in brain tissues, including microglia and endothelial cells [63,64]. ICAM and the Selectins are important regulators of cell–cell interaction and known to be involved in metastasis and angiogenesis [63,64]. ICAM-1 has been shown to promote inflammatory responses during HIV infection, leading to accelerated disease progression [65]. Taken together, these findings underscore the potent role of Tat-mediated inflammation across different cell types.

Inflammatory responses are largely initiated in response to TLR activation [66,67]. To understand the mechanism of Tat-induced inflammation in TZM-bl cells, we examined the expression of various TLRs and the potential intermediates involved. Among other TLRs, we observed increased expression of TLR7, along with IRAK4 and NF-κB, in TZM-bl cells post Tat-stimulation. TLR-mediated immune dysregulation has been reported in T cells, pDCs, and monocytes [68,69] during HIV immunopathogenesis, and blocking TLR7 has been shown to reduce immune activation [70,71]. In a murine model, HIV-1 ssRNA has been shown to elevate pro-inflammatory cytokines through TLR7 activation [72]. HIV proteins have also been shown to trigger cytokine production through distinct TLR signaling pathways in various cell types. HIV-1 structural proteins (p17, p24, and gp41) increased IL-8 by activating TLR2 [73] and TLR10 [74] and involving the NF-ĸB axis in primary T cells, as well as TZM-bl cells. Gp120 stimulated TLR-2 and TLR-4 mediated induction of TNF-α and IL-8 via activation of NF-κB in the primary genital epithelial cells [54]. Unlike other HIV proteins, less is known about the role of the Tat protein in modulating TLR-mediated responses. A single study shows that Tat directly binds to TLR4 and its co-receptor MD-2, leading to the induction of pro-inflammatory cytokines such as TNF-α and IL-10 [75], contributing to immune dysregulation in immune cells. TLR signaling pathways, including TLR7, culminate in the activation of NF-κB, a transcription factor crucial for inducing inflammation and other immune responses and regulating HIV disease progression [76,77,78]. Evidence indicates that Tat modulates key enzymes in NF-κB signaling. HIV-Tat interacts with IκB-α and p65, thereby increasing NF-κB transcriptional activity in HeLa cells. Tat has also been shown to inhibit SIRT-1, a negative regulator of T cell activation, leading to hyperimmune activation [79,80]. In corroboration, we also noted upregulation of NF-ĸB in response to HIV-Tat in the TZM-bl cells. Further blocking of NF-ĸB using BAY 11-7082 (40 µM) significantly decreased the expression of pro-inflammatory cytokines (TNF-α, IL-1β, and IFN-β) (Figure S5). These findings suggest that NF-κB is a crucial factor driving inflammatory responses in cervical cells. Besides NF-ĸB, Tat stimulation also increased the expression of IRAKs, a crucial component of TLR-mediated NF-ĸB activation and also reported during HIV immunopathogenesis. Although we did not observe a significant change in the expression of IRAK1 in Tat-stimulated TZM-bl cells. In a previous study carried out on HIV infected microglial cells it was observed that IRAK blocking led to induce a pro-inflammatory milieu, highlighting the role of TLR-7 and TLR-8 [81]. We also observed that, upon inhibiting the TLR7 by its inhibitor (M5049), the expression of intermediate molecules NF-ĸB, IRAK-1/-4 and cytokine IFN-β was significantly reduced. Collectively, these findings underscore that TLR7-mediated inflammatory responses might be a critical factor during cervical inflammation; however, the present study could not establish a direct link between TLR7 to Tat-stimulated inflammation, which needs detailed investigations.

Cervical inflammation not only speeds up the disease progression in HIV infected women, decreases the efficacy of the local antiretrovirals, but can also influence EMT, leading to cancer progression, particularly in HIV-HPV co-infected women. Hence, understanding the factors involved in regulating inflammation is warranted. miRNAs are one such regulator, known to mitigate the inflammatory processes. The role of miRNA-204-5p has been reported in various inflammation-associated conditions, including neuroinflammation, corneal inflammation, and synovial inflammation, among others [32,49,82,83]. In microglial cells, miR-204-5p has been shown to suppress Tat-mediated neuroinflammation through ferroptosis [30,32]. In line with previous reports, we also observed downregulation of miR-204 in TZM-bl cells, along with increased levels of inflammatory cytokines, in response to Tat stimulation. Using the gain-of-function approach—i.e., transfecting the cells with a specific mimic—we further showed that overexpression of miR-204-5p indeed suppressed the pro-inflammatory mediators, such as TNF-α, IL-6, MIP-1α, MIP-1β, ICAM-1, and P-Selectin, as well as ROS. miR-204-5p has been previously shown to regulate oxidative stress associated with neuroinflammatory changes and depression phenotypes in the murine model by targeting via the NF-ĸB axis RGS12 [32]. miR-204-5p also regulates myopia development by targeting TXNIP [84]. MiRNAs exert their regulatory effect by targeting specific proteins. Several reports show that miR-204-5p suppresses NF-ĸB activity [31,32]. We noted an inverse profile of miR-204 and inflammatory mediators—i.e., cytokines and NF-ĸB—upon Tat stimulation, indicating their probable association. We confirmed these observations by overexpressing miR-204-5p in TZM-bl cells, which resulted in reduced NF-ĸB and inflammatory cytokine expression after Tat stimulation. Using miRWalk, an in silico tool, we predicted that the putative target site of miRNA-204-5p was located within the 3′UTR of NF-κB, which we further validated using a luciferase assay, underscoring that miR-204-5p targets the NF-κB axis in the regulation of inflammatory responses.

A dynamic interplay reportedly occurs between NF-κB signaling and reactive oxygen species, and ROS-mediated HIV LTR activation through NF-κB has been reported in Jurkat cells [85]. HIV-Tat has been shown to potentiate NF-κB-induced inflammatory responses by altering cellular redox state in HeLa cells [86,87]. We also observed Tat-induced ROS and NF-κB upregulation in TZM-bl cells, which was downregulated in miR-204-5p-transfected cells. Together, these findings reveal that oxidative stress production and NF-κB activation are interlinked in Tat-mediated cervical inflammatory responses. However, their regulatory roles need to be explored further. Previous studies revealed that Tat activates NF-κB in HEK, HL3T1, and Jurkat cells, among others [12,88]. In line with these studies, we also observed NF-κB activation in Tat-stimulated TZM-bl cells. Nuclear translocation of phospho-NF-κB was increased in Tat-stimulated TZM-bl cells. It was also noted that miR-204-5p mimic reduced the nuclear localization of phospho-NF-κB in these Tat-stimulated cells. In contrast, miR-204-5p inhibitor increased phospho-NF-κB in TZM-bl cells upon Tat stimulation. These results indicate that NF-κB activation in Tat-stimulated TZM-bl cervical cells is miR-204-5p dependent manner.

**Figure 6:**
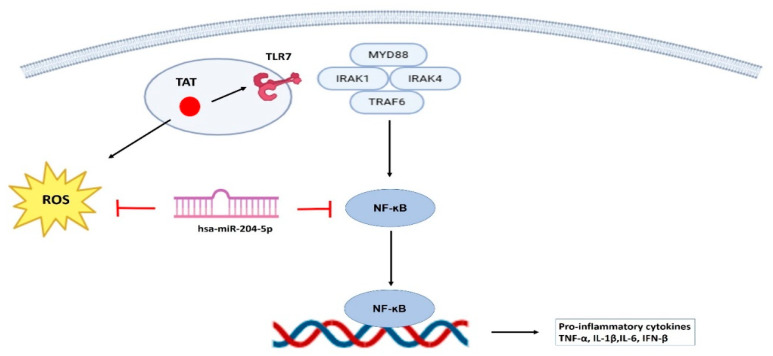
Schematic showing the mechanism involved in HIV-1 Tat-mediated cervical inflammation. HIV-1 Tat enhances inflammatory responses in TZM-bl cervical epithelial cells via activation of the TLR7/NF-κB axis, where miR-204-5p emerges as a key regulatory molecule by targeting NF-κB, thereby attenuating the expression of pro-inflammatory cytokines. In addition, miR-204-5p reduces ROS levels in Tat-stimulated TZM-bl cells, highlighting its potential role in modulating cervical inflammation and mucosal immunity.

Our study provides mechanistic insights into HIV-1 Tat-induced inflammatory processes in the cervical cells. In previous studies, we [89] and others [2,90,91,92] have reported elevated cervical inflammation in cytobrush-derived cervical cells and also in cervicovaginal lavage samples of HIV-infected women. However, the regulatory mechanisms driving inflammation in cervical cells remain poorly understood. A major challenge in studying these mechanisms is the limited availability of cervical cells and the unavailability of suitable animal models. To overcome these limitations and gain detailed molecular insights, TZM-bl cells have been widely used as an in vitro model for cervical studies [93,94], which was also used in the current exploratory study. However, our findings are based solely on in vitro assays using TZM-bl cells, which is one of the study’s limitations. TZM-bl cells are transformed cancer-derived epithelial cells that may not fully represent the physiological state of normal cervical epithelial cells. Further, these cells lack the cellular diversity of the cervicovaginal mucosa; hence, the findings presented here need to be confirmed using a primary cervical cell line, cervical explant culture and/or in vivo models. To the best of our knowledge, this exploratory study is the first to provide evidence that HIV-1 Tat increases inflammation in cervical epithelial cells (TZM-bl) via the miR-204-5p/NF-κB axis (Figure 6). The mechanism of the upstream of NF-κB cascading and TLR7 activation by HIV-1 Tat protein needs to be explored further. Our findings offer functional insights into the interaction between HIV-1 Tat and the miR-204-5p/NF-κB signaling axis, underscoring its possible relevance to cervical inflammation and mucosal immunity, which needs further investigation.

## Figures and Tables

**Table 1:** The sequences of PCR primers and restriction enzymes used in genotyping analysis.

Genes	Primer Sequence	Reference
β-actin	F-5′-TCGTCCACCGCAAATGCTTCTAG-3′ R-5′-ACTGCTGTCACCTTCACCGTTCC-3′	[41]
IL-1β	Fq-5′-ATGCACCTGTACGATCACTG-3′ Rq-5′-ACAAAGGACATGGAGAACACC-3′	[42]
IL-6	Fq-5′ACCCCCAATAAATATAGGACTGGA-3′ Rq-5′-GCTTCTCTTTCGTTCCCGGT-3′	[43]
TNF-α	Fq-5′-CTGGGGCCTACAGCTTTGAT-3′ Rq-5′-GGCTCCGTGTCTCAAGGAAG-3′	[44]
IFN-β	F-5′-GGTTACCTCCGAAACTGAAGA-3′ R-5′-CCTTTCATATGCAGTACATTAGCC-3′	[45]
NF-κB	Fq-5′-TCTCCCTGGTCACCAAGGAC-3′ Rq-5′-TCATAGAAGCCATCCCGGC-3′	[46]
IRAK1	5′-ACGGACACCTTCAGCTTTGG-3′ 5′-TCCACCAGGTCTTTCAGATACTTG-3′	# (NM_001025242.2)
IRAK4	F-5′-TCATAGGCGGCAGGAACTTA-3′ R-5′-ACCCAAACACTTCCCATCAG-3′	# (NM_001145257.2)
TLR7	F-5′-GTTACCAGGGCAGCCAGTTC-3′ R-5′-ATGAGCCTCTGATGGGACAA-3′	[47]

(#—primer designed from the NCBI database using the NCBI reference sequence).

## Data Availability

Data are contained within the results section of this article.

## Supplementary Materials

The following supporting information can be downloaded at https://www.mdpi.com/article/10.3390/cells15020117/s1. Figure S1. Heat-inactivated Tat protein was used as a negative control in addition to unstimulated cells. Expression of TLR7, NF-kB and IRAK4 was assessed by real-time PCR. Statistical analysis was performed by using the ANOVA followed by post hoc Dunnett’s test. * indicates *p* < 0.05. Figure S2. Validation of miR-204-5p upregulation in TZM-bl cells upon miR-204-5p mimic treatment. TZM-bl cells were transfected with 50 nM of miR-204-5p mimic, or scrambled miRNA mimic (mock) for 24 h. Expression of the miR-204-5p gene was assessed by real-time PCR. Statistical analysis was performed by using the unpaired *t*-test. **** indicates *p* < 0.0001. Figure S3. The effect of miR-204-5p inhibitor (50nM) was checked in Tat-stimulated TZM-bl cells. TZM-bl cells were transfected with 50 nM of miR-204-5p inhibitor or scrambled miRNA mimic (mock) for 24 h. Protein expressions of NF-κB, IRAK-1 and IL-1β were assessed by Western blotting. Statistical analysis was performed by using the ANOVA followed by post hoc Dunnett’s test. *p*-value for all the conditions was >0.05. Figure S4. The effect of the TLR7 inhibitor, M5049, was assessed on the mRNA expression of inflammatory cytokines and signaling pathway mediators. TZM-bl cells were treated with M5049 (25 nM) one hour before Tat treatment. The gene expression profiles of IFN-β, NF-κB, IRAK1, and IRAK4 were analyzed by real-time PCR. Fold change mRNA expression was calculated over the respective control. Statistical analysis was performed by using the one-way ANOVA followed by post hoc Dunnett’s test. * indicates *p* < 0.05, ** indicates *p* < 0.01, *** indicates *p* < 0.001. Figure S5. The effect of the NF-κB inhibitor, Bay 11-7082, was checked on mRNA expression of inflammatory cytokines. TZM-bl cells were treated with Bay 11-7082 (40 µM) one hour before Tat treatment. The gene expression profile of TNF-α, IL-1β, IFN-β and IL-6 was analysed by Real Time-PCR. Fold change mRNA expression was calculated over non-stimulated cells. Statistical analysis was performed by using the one-way ANOVA followed by post hoc Dunnett’s test. * indicates *p* < 0.05, ** indicates *p* < 0.01, **** indicates *p* < 0.0001.
