# Supplementary material for: miR-204 Negatively Regulates HIV-Tat-Mediated Inflammation in Cervical Epithelial Cells via the NF-κB Axis: Insights from an In Vitro Study
Source: Cells. 2026 Jan 9;15(2):117. doi: 10.3390/cells15020117 (PMC12839329; doi:10.3390/cells15020117)
Supplement: Supplementary file 1 [file cells-15-00117-s001.zip › cells-3807267-supplementary.pdf]

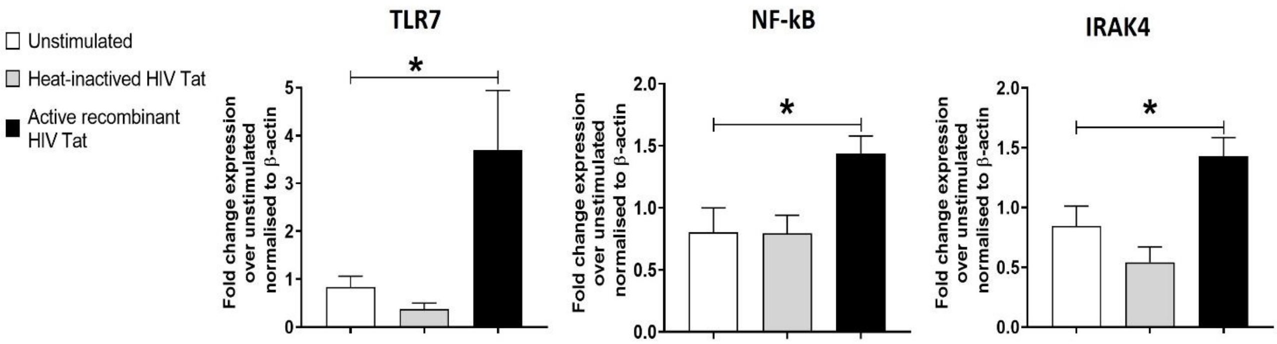

Figure S1. Heat-inactivated Tat protein was used as a negative control in addition to unstimulated cells. Expression of TLR7, NF-kB and IRAK4 was assessed by real-time PCR. Statistical analysis was performed by using the ANOVA followed by post hoc Dunnett's test. *indicates $p<0.05$.

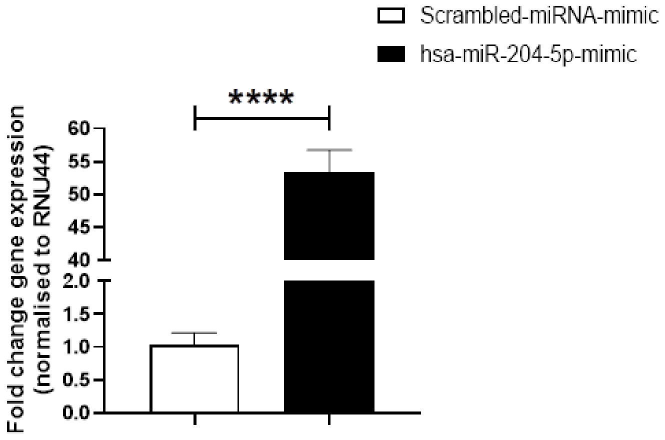

Figure S2. Validation of miR-204-5p upregulation in TZM-bl cells upon miR-204-5p mimic treatment. TZM-bl cells were transfected with 50 nM of miR-204-5p mimic, or scrambled miRNA mimic (mock) for 24 h. Expression of the miR-204-5p gene was assessed by real-time PCR. Statistical analysis was performed by using the unpaired t-test **** indicates $p<0.0001$.

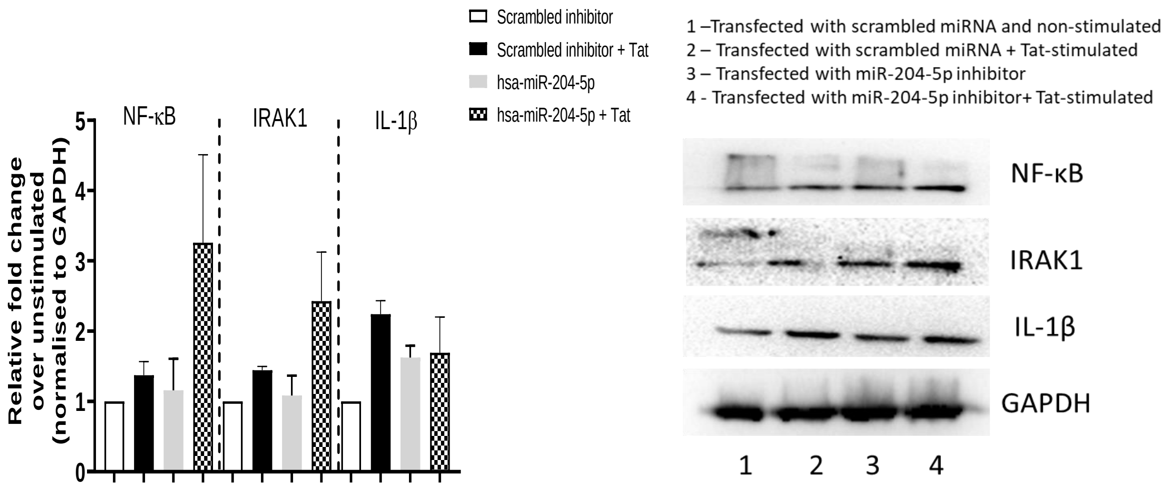

Figure S3. The effect of miR-204-5p inhibitor (50nM) was checked in Tat-stimulated TZM-bl cells. TZM-bl cells were transfected with 50 nM of miR-204-5p inhibitor or scrambled miRNA mimic (mock) for 24 h. Protein expressions of NF-κB, IRAK-1 and IL-1β were assessed by Western blotting. Statistical

analysis was performed by using the ANOVA followed by post hoc Dunnett's test. *p*-value for all the conditions was >0.05.

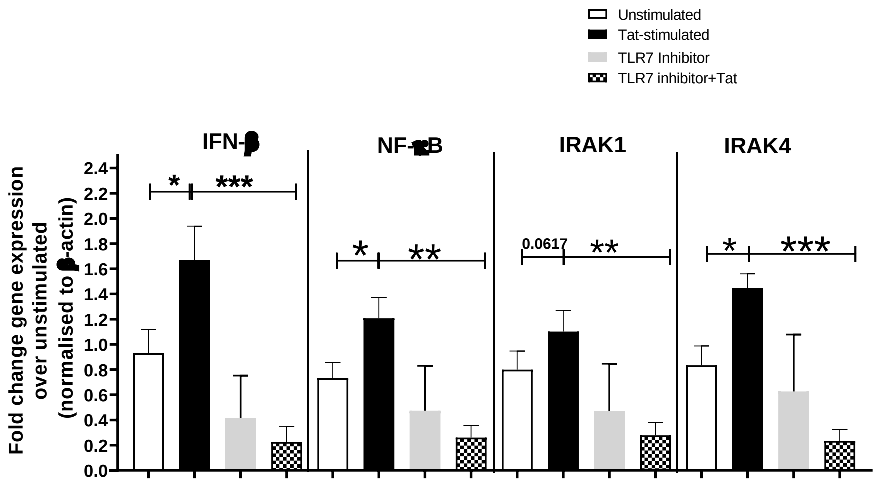

Figure S4. The effect of the TLR7 inhibitor, M5049, was assessed on the mRNA expression of inflammatory cytokines and signaling pathway mediators. TZM-bl cells were treated with M5049 (25 nM) one hour before Tat treatment. The gene expression profiles of IFN-β, NF-κB, IRAK1, and IRAK4 were analyzed by real-time PCR. Fold change mRNA expression was calculated over the respective control. Statistical analysis was performed by using the one-way ANOVA followed by post hoc Dunnett's test. * indicates $p<0.05$, ** indicates $p<0.01$, *** indicates $p<0.001$.

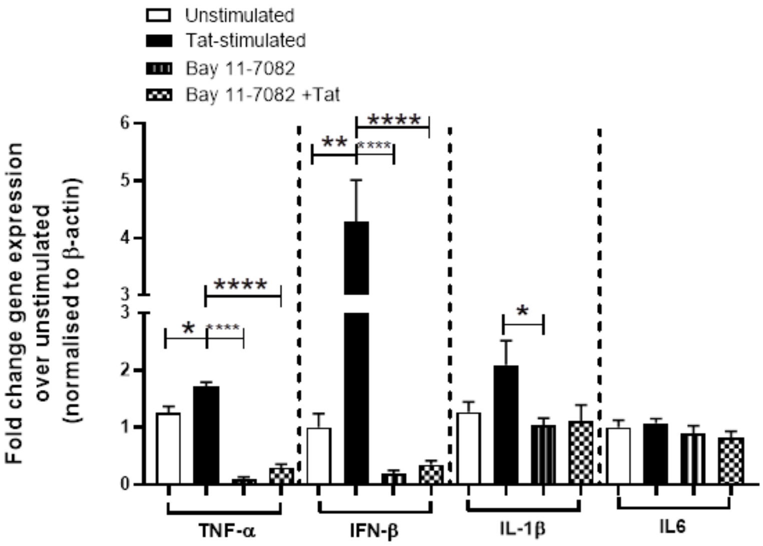

Figure S5. The effect of the NF-κB inhibitor, Bay 11-7082, was checked on mRNA expression of inflammatory cytokines. TZM-bl cells were treated with Bay 11-7082 (40 μM) one hour before Tat treatment. The gene expression profile of TNF-α, IL-1β, IFN-β and IL-6 was analysed by Real Time-PCR. Fold change mRNA expression was calculated over non-stimulated cells. Statistical analysis was performed by using the one-way ANOVA followed by post hoc Dunnett's test. *indicates $p<0.05$, ** indicates $p<0.01$, **** indicates $p<0.0001$.
